# Development of low back pain curriculum content standards for entry-level clinical training

**DOI:** 10.1186/s12909-024-05086-x

**Published:** 2024-02-12

**Authors:** Hazel J. Jenkins, Benjamin T. Brown, Mary O’Keeffe, Niamh Moloney, Chris G. Maher, Mark Hancock

**Affiliations:** 1 Faculty of Medicine, Health and Human Sciences, Macquarie University, Sydney, Australia; 2 Institute for Musculoskeletal Health, The University of Sydney, Sydney Musculoskeletal Health, Sydney, Australia; 3 Faculty of Health Sciences, Curtin University, Perth, Australia; 4 Sydney Musculoskeletal Health, The University of Sydney, Sydney, Australia

**Keywords:** Low back pain, Entry-level clinical training, Curriculum content standards, Medical education, Healthcare education

## Abstract

**Background:**

The management of low back pain (LBP) is highly variable and patients often receive management that is not recommended and/or miss out on recommended care. Clinician knowledge and behaviours are strongly influenced by entry-level clinical training and are commonly cited as barriers to implementing evidence-based management. Currently there are no internationally recognised curriculum standards for the teaching of LBP content to ensure graduating clinicians have the appropriate knowledge and competencies to assess and manage LBP. We formed an international interdisciplinary working group to develop curriculum content standards for the teaching of LBP in entry-level clinical training programs.

**Methods:**

The working group included representatives from 11 countries: 18 academics and clinicians from healthcare professions who deal with the management of LBP (medicine, physiotherapy, chiropractic, osteopathy, pharmacology, and psychology), seven professional organisation representatives (medicine, physiotherapy, chiropractic, spine societies), and one healthcare consumer. A literature review was performed, including database and hand searches of guidelines and accreditation, curricula, and other policy documents, to identify gaps in current LBP teaching and recommended entry-level knowledge and competencies. The steering group (authors) drafted the initial LBP Curriculum Content Standards (LBP-CCS), which were discussed and modified through two review rounds with the working group.

**Results:**

Sixty-two documents informed the draft standards. The final LBP-CCS consisted of four broad topics covering the epidemiology, biopsychosocial contributors, assessment, and management of LBP. For each topic, key knowledge and competencies to be achieved by the end of entry-level clinical training were described.

**Conclusion:**

We have developed the LBP-CCS in consultation with an interdisciplinary, international working group. These standards can be used to inform or benchmark the content of curricula related to LBP in new or existing entry-level clinical training programs.

**Supplementary Information:**

The online version contains supplementary material available at 10.1186/s12909-024-05086-x.

## Background

Low back pain (LBP) is a common condition and the leading cause of years lived with disability worldwide [1]. While individual episodes of LBP may resolve quickly with minimal intervention, LBP recurrence and the development of persistent pain are also common and contribute to the overall healthcare burden associated with LBP [2, 3]. Clinical practice guidelines are available to guide the appropriate management of LBP and improve patient outcomes [4, 5]. Despite these recommendations, however, the management of LBP is highly variable [6]. Many patients receive management that is not recommended in current guidelines (e.g., imaging, opioids) and/or miss out on the care that is recommended (e.g., education, exercise). Both these problems may lead to poorer patient outcomes [4, 6].

Strategies to educate clinicians and implement best-evidence and guideline recommendations into clinical practice have been attempted, with little current evidence of success [7]. Researchers have identified that a clinician’s beliefs, perceptions and level of clinical knowledge may influence the uptake of LBP guideline recommendations into clinical practice [8]. In particular, the influence of formal entry-level clinical training has been highlighted as a potential barrier to the uptake of clinical practice guidelines for LBP [8, 9], and conversely, that changing student beliefs and attitudes about LBP in clinical training programs may facilitate more guideline-adherent practice in future clinicians [10].

Entry-level clinical training programs refer to undergraduate or postgraduate programs that train clinicians to enter healthcare professions [11]. Education related to LBP is variable across training programs, both within and between different healthcare professions. The time spent delivering LBP or general pain management content varies considerably across different clinical training programs [9, 12–16]. Furthermore, gaps have been identified in different clinical training programs with regards to student knowledge and competencies related to LBP [10, 17–22], confidence in ability to manage LBP on graduation [23–25], and alignment with LBP clinical guideline recommendations [12, 26].

Appropriate LBP curriculum content within entry-level clinical training programs is needed to ensure that healthcare professionals are graduating with the knowledge and skills needed to deliver high-quality evidence-based care in clinical practice. Curriculum content standards are defined as the curriculum needed to equip clinicians with the knowledge, skills and attitudes necessary at the time of graduation [27]. Currently, while core competencies for clinical training programs as a whole have been developed [28], there are no specific content standards to guide teaching for LBP. Therefore, we aimed to develop the first curriculum content standards for the teaching of LBP in entry-level clinical training programs worldwide.

## Methods

### Overview and scope of the development of the low back pain curriculum content standards

An international, interdisciplinary working group, led by a steering group (authors), was formed to develop the Low Back Pain Curriculum Content Standards (LBP-CCS) using an iterative process. An initial literature review was performed by the steering group to identify the range of content to be included in the LBP-CCS. The content and structure of the LBP-CCS was then modified through two rounds of group discussion and feedback from the working group. The final version of the LBP-CCS was approved by all members of the working group. Ethical approval was not required for the development of the LBP-CCS as no participants or participant data were recruited or collected. All members of the working group who contributed to the LBP-CCS are acknowledged in this publication.

The LBP-CCS were developed to include a complete list of content items necessary for comprehensive education on LBP epidemiology, diagnosis, and management. Input into the development was sought from a diverse range of healthcare professions involved in the management of LBP. The working group recognised that different healthcare professions may require different levels of knowledge related to the diagnosis and management of LBP. Therefore, the LBP-CCS were designed to provide guidance that can be implemented to the appropriate level for individual entry-level clinical training programs.

### Formation of the working group

The steering group (authors) identified professional organisations, academics, researchers, clinicians, and consumers to invite to participate in the development of the LBP-CCS. International professional organisations with interest in the management of LBP in primary care were invited to be involved in the development of the LBP-CCS. Organisations agreeing to be involved were asked to nominate a representative to be part of the working group. Other potential working group members were purposively invited to achieve a spread of different occupational and clinical backgrounds, sex, and geographic location.

Of 15 organisations approached, seven agreed to participate in the LBP-CCS development and provide representatives to join the working group. Participating organisations included: International Society for the Study of the Lumbar Spine (ISSLS), International Federation of Orthopaedic Manipulative Physical Therapists (IFOMPT) on behalf of World Physiotherapy (WP), World Federation of Chiropractic (WFC), European Pain Federation (EFIC), Musculoskeletal Association of Chartered Physiotherapists (MACP), and Council of Physiotherapy Deans Australia and New Zealand (CPDANZ). Responses were not received from the remaining organisations approached, which included invitations to medical and osteopathic organisations. A further 22 academics, researchers, clinicians, or healthcare consumers were invited to join the working group, with 19 accepting, leading to a final working group of 32 participants (including the steering group). The spread of occupational backgrounds, sex, and geographic locations represented within the working group is presented in Table 1.

**Table 1:** Characteristics of the members of the Low Back Pain Curriculum Content Standards working group (including steering group)

Working group characteristic	Number of participants (%)
Professional background*	
Academic/researcher Clinician Organisation representative Healthcare consumer	28 (85%) 9 (27%) 7 (21%) 1 (3%)
Clinical background*	
Physiotherapy Chiropractic Medicine Osteopathy Pharmacy Psychology	18 (55%) 9 (27%) 4 (12%) 1 (3%) 1 (3%) 1 (3%)
Sex	
Male Female	18 (55%) 15 (45%)
Geographic location	
Australia and New Zealand Europe North America Africa Asia	14 (42%) 10 (30%) 7 (21%) 1 (3%) 1 (3%)

*Members of the working group may represent more than one professional or clinical background

%: Percentage

### Literature review to inform development of the low back pain curriculum content standards

The steering group conducted an initial review of the literature to establish a draft list of content to be included in the LBP-CCS. Three search strategies were used to find relevant literature:

1. Medline (OVID), Embase (OVID), CINAHL, and PsycInfo were searched from inception to March, 2022 to identify current gaps in entry-level clinical education related to LBP. Search terms related to: (i) LBP; (ii) curriculum/knowledge; and (iii) healthcare students. Searches were developed for each database and are available in Additional file 1. Articles were screened by one member of the steering group (HJ) and were included if the article assessed or discussed LBP or pain education in an entry-level clinical training program. ‘Education’ could relate to any of the following: required competencies, learning outcomes, identified gaps, student preparedness for clinical practice, or alignment with evidenced-based practice or clinical practice guidelines. Articles discussing clinical practice with respect to the required competencies or knowledge needed from entry-level clinical training were also included. Clinical training programs could relate to any healthcare profession that requires training in LBP epidemiology, diagnosis, or management.
2. Clinical practice guidelines and accreditation documents, identifying required competencies or knowledge for healthcare clinicians related to the management of LBP, were identified by the steering group. To be included, clinical practice guidelines needed to be related to the multidisciplinary management of LBP in primary care, be produced by a national organisation, and be informed by literature review. A published overview of clinical practice guidelines [5] meeting these criteria was used to identify guidelines for inclusion. A search was performed for updates to the guidelines identified in the overview, with the most recent version included. National and international accreditation documents were included if they related to entry-level clinical training programs in medicine, physiotherapy, or chiropractic, and were written in English. Summary documents, collating information across multiple clinical guidelines or accreditation documents, were used where available.
3. The working group was asked to recommend documents, including curriculum and policy documents and new or updated guidelines not captured by the above process, that they considered appropriate to inform the development of the LBP-CCS.

From each included article or document, one of the steering group (HJ, BB, MO, MH) extracted the key findings, gaps, or requirements related to LBP education that were identified and categorised as content/knowledge and skills/competencies required.

### Iterative development of the low back pain curriculum content standards

The first iteration of the LBP-CCS was developed by the steering group. The extracted data from the literature review were collated by one of the steering group (HJ) under broad topic headings. These topic headings were then discussed with the members of the steering group to determine an initial topic structure. The extracted data were then collated into the topic structure, with consolidation of individual items where there was duplication of data. It was not considered within the scope of the development of the LBP-CCS to evaluate the strength of available evidence and provide specific recommendations on how the content should be taught. Instead, the aim of the LBP-CCS was to provide high-level guidance of the content topics to be included within curriculum for LBP and be taught within an evidenced-based framework.

Two rounds of review, including group discussion and written feedback, were held with the working group to determine any necessary changes to the draft LBP-CCS. For each review round, members of the working group were provided with the latest iteration of the LBP-CCS and a feedback document, including the questions to be reviewed within group discussion and opportunity to provide more specific written feedback on each element of the LBP-CCS. Multiple online discussion groups were held to accommodate time-zone differences and enable all working group members to attend a session. Each discussion group was recorded (with permission of the working group members in attendance) and had at least two of the steering group members in attendance, to moderate the group discussion and record notes. The review questions were discussed within each group. Key discussion points from groups were also presented at subsequent groups within the same round to encourage further discussion. After each of the review rounds, both the feedback from the discussion groups and feedback documents were collated, qualitatively summarised, and a list of potential changes developed and discussed within the steering group. Where feedback was conflicting, potential changes were suggested in alignment with the majority of opinions from the working group and flagged for discussion within the next working group review. For each new iteration of the LBP-CCS, a summary of the changes was provided to the working group and discussed within the following review round. In this way, the working group were able to provide feedback on the changes which were incorporated into the following review round.

## Results

### Literature review

The database search returned 577 articles, of which 57 were screened for full-text and 34 were included for data extraction. A previously published paper summarising 15 clinical practice guidelines from Africa, Australia, Brazil, Belgium, Canada, Denmark, Finland, Germany, Malaysia, Mexico, the Netherlands, Philippine, Spain, the USA, and the UK was used to extract clinical guideline recommendations [5]. Updates to two of the 15 guidelines were identified and used to extract guideline recommendations [29, 30]. Ten accreditation documents were sourced from international and regional (North America, Australasia, Europe) accrediting bodies for medical, physiotherapy, and chiropractic entry-level clinical training programs. On assessing these accreditation documents, we decided not to source accreditation documents from other healthcare professions, as no criteria or competencies specific to LBP were found within the sourced documents. Seventeen additional documents were identified by the working group including clinical care standards, a musculoskeletal education framework, curriculum documents, and LBP overview papers. No new or updated clinical practice guidelines were identified by the working group. The complete list of documents used to inform the development of the LBP-CCS is available in Additional file 2.

### Iterative development of the low back pain curriculum content standards

#### First iteration

Data from the literature review were collated under 12 topic headings as described in Table 2. The steering group determined the structure of the first iteration to include: (i) the overarching objectives of the LBP-CCS; and (ii) 10 topic headings outlining the content to be included (Table 2). The individual content items were listed under: (i) principles; (ii) knowledge; and (iii) skills. The sub-heading ‘principles’ was intended to capture context to clarify the intent of the required knowledge and skills for each topic. For example, under the topic ‘Investigations’ one of the principles was for clinicians to consider whether investigation findings will substantially alter patient management; whereas, the associated knowledge item required that clinicians know the risks and benefits of the proposed investigations. The related skills item in this example stated that clinicians should be able to order and interpret investigations appropriately.

**Table 2:** Topic headings and number of included individual content items in each iteration of the Low Back Pain Curriculum Content Standards

Data collation (number of items)	Iteration 1 (number of items)	Iteration 2 (number of items)	Iteration 3 (number of items)
Basic/clinical sciences (10)	Objectives (6)	Preamble (1)	Preamble (1)
Epidemiology of low back pain (13)	Basic/clinical sciences (9)	Objectives (6)	Objectives (6)
Biopsychosocial model/Person-centered approach (9)	Epidemiology of low back pain (12)	Epidemiology of low back pain and the public health impact (7)	Epidemiology of low back pain and the public health impact (12)
Assessment of low back pain (21)	Biopsychosocial model/Person-centered approach (7)	Biopsychosocial factors contributing to the development and prognosis of low back pain (9)	Biopsychosocial contributors to the development and course of low back pain (10)
Investigations (7)	Diagnosis/Conditions (11)	Low back pain diagnosis and classification (8)	Clinical assessment and investigations (15)
Diagnosis/Conditions (21)	Clinical assessment (8)	Clinical assessment (8)	Developing a clinical management plan for low back pain (17)
Guideline recommendations (3)	Investigations (6)	Investigations (7)	Glossary (24)
Management (46)	Management (15)	Low back pain management and prevention (17)	Suggested resources (9)
Referral/Collaboration (17)	Referral/Collaboration (4)	Suggested resources (4)	
Prevention (2)	Prevention (4)		
Outcomes (5)	Outcomes/Reassessment (3)		
Teaching styles/modalities (4)			

#### Second iteration

The first round of review with the working group was used to inform the second iteration of the LBP-CCS. The first review round focused on: (i) the appropriateness of the topic structure; (ii) the level of detail included within the content items and whether more specific recommendations should be made; (iii) the inclusion of content items not specific to LBP education (e.g., communication skills, clinical reasoning); and (iv) specific feedback on the individual content items or suggestions for additional/missing content items. The general structure of the document was agreed to be appropriate; however, a preamble to provide context to the document was thought necessary and suggestions were made to integrate some of the existing topic headings to improve the flow of the document and reduce repetition (Table 2). While the separation of ‘Principles’, ‘Knowledge’, and ‘Skills’ under each topic heading was considered important, the working group thought that ‘Principles’ should be replaced with an explanatory statement under each topic heading to explain alignment within the current evidence-base. The working group preferred the term ‘Competency’ to ‘Skills’ to reflect the move of many academic programs to competency-based teaching and assessment.

The working group agreed that the LBP-CCS should provide the general topics of content to be included (e.g., the risks and benefits of management options for LBP) rather than provide the specific evidenced-based recommendations (e.g., opioids should not be used in the management of LBP). This was to ensure that the LBP-CCS would be appropriate to use across different entry-level training programs and that the LBP-CCS would not become out-dated as new evidence becomes available. It was considered important, however, that the preamble clearly outline the need to apply the LBP-CCS within an evidenced-based context as appropriate for the clinical training program and local context/culture. A ‘Suggested resource’ section was also recommended to provide current evidence-based resources that could be used to inform application of the LBP-CCS. Regular review and update of the LBP-CCS (e.g., every five-years) was recommended to ensure that the standards align with emerging research findings. The inclusion of items not specific to LBP education but important in the development of appropriate patient management (e.g., patient communication, clinical reasoning), were considered essential. However, it was suggested that these be integrated within the items specific to LBP rather than included as stand-alone content items (e.g., ‘Synthesise clinical assessment findings and communicate a meaningful explanation of their LBP to the patient’).

Finally, feedback related to the specific content items was incorporated into the second iteration of the LBP-CCS. This included the addition of new content items and the removal/rewording of some content items to limit repetition, increase the consistency of language throughout the document, and increase the focus on some content items.

#### Third iteration

The second round of review with the working group was used to inform the third, and final, iteration of the LBP-CCS. The second review round focused on: (i) the appropriateness of the new sections of the LBP-CCS (preamble, explanatory statements, suggested resources); (ii) the structure/flow of the topic headings and included content items; and (iii) specific feedback on the individual content items. Overall, there was support for the new sections of the LBP-CCS, with some minor changes or additional resources suggested. Within the discussion groups it was highlighted that there were some differences in the interpretation of terms/words between members of the working group. The addition of a glossary to define common terms within the document was recommended. The topic flow was considered improved from the first iteration; however, to further improve the flow, it was suggested that the ‘Clinical assessment’ and ‘Investigations’ topics be collapsed together, and to integrate the ‘Low back pain diagnosis and classification’ topic across the remaining topics. The final topic structure is presented in Table 2. The third iteration of the LBP-CCS was approved for dissemination and implementation by all members of the working group. The final LBP-CCS is available in Additional file 3.

## Discussion

### Key findings

We have developed curriculum content standards for LBP education in entry-level clinical training programs. The content items included in the LBP-CCS were informed by current literature, clinical practice guidelines, accreditation requirements, and other policy documents. The structure and content of the LBP-CCS were reviewed through three iterations and approved by an interdisciplinary international working group. The developed LBP-CCS are ready to be implemented in entry-level clinical training programs to inform the development or review of LBP curriculum and ensure that graduates have the knowledge and competencies required to deliver high-quality care to patient with LBP in clinical practice. The LBP-CCS will be reviewed and updated periodically to ensure that it remains current.

### Comparison to previous literature

To our knowledge, curriculum content standards for LBP entry-level clinical training have not been previously developed. Current clinical practice guidelines [5] and clinical care standards for LBP [6] that exist have been developed to inform clinical practice for qualified clinicians with existing knowledge about LBP. Instead, we developed the LBP-CCS to focus on the curriculum requirements for entry-level clinical students with no prior knowledge of LBP. For example, clinical guidelines tend to focus on the appropriate assessment and management of LBP [5] and do not provide details of required knowledge related to the epidemiology and course of LBP that underpins clinical reasoning and management decisions. A similar outline of recommended curriculum content in healthcare programs has been developed for pain education as a whole (IASP Interprofessional Pain Curriculum Outline) [31]; however, this does not include details specific to LBP that are important to highlight within entry-level clinical training. For example, imaging is rarely recommended in the assessment of LBP and inappropriate use has been associated with poorer patient outcomes [32]; details such as determining the appropriate use of imaging can be highlighted more specifically in the LBP-CCS rather than within curriculum content for general pain [31], where the concept may not be relevant for all pain presentation types.

### Strengths and limitations

A systematic and rigorous approach was used to develop the LBP-CCS. The working group was selected to ensure representation across diverse healthcare professions involved in the management of LBP, geographic locations, and professional backgrounds with academic, clinician, and consumer involvement. Eleven countries across 5 continents were represented within the working group; however, there was an underrepresentation of developing countries (1/11, 9%). Similar concerns related to the implementation of best-practice care for LBP have been identified globally [4], and, therefore, similar education requirements are likely to be needed. However, curriculum content requirements for developing countries may not have been completely explored. We therefore recommend, in the preamble to the LBP-CCS, that the LBP-CCS be implemented with consideration of the local context and environment. Physiotherapists and chiropractors commonly manage patients with LBP in primary care, which is reflected in more hours on LBP education in entry-level clinical training programs [12]. Therefore, we included larger proportions of physiotherapists and chiropractors within the working group to ensure that the LBP-CCS reflected the content required by programs with a stronger focus on LBP education. Moving forward, we intend to develop modified versions of the LBP-CCS for healthcare professions that are involved in the management of LBP but have different educational needs, such as medicine, pharmacy, clinical psychology, clinical exercise physiology, occupational therapy, and nursing. The current version can still be used to inform the education of all health professionals who treat people with LBP, but individual programs will need to consider the level of detail required.

The first iteration of the LBP-CCS was informed by a review of the literature and other professional policy documents. The literature search was performed in March, 2022 and new literature or guideline documents may change the content of the LBP-CCS. To minimise this limitation, regular review of the literature is planned by the steering group to ensure that the LBP-CCS remain current. The working group did not identify any new or updated clinical practice guidelines during the development process; however, the World Health Organization have published new guidelines for the management of chronic LBP since the development process concluded (December, 2023) [33]. The new guidelines have been assessed by the steering committee, and the guideline messages are consistent with the LBP-CCS. A sparsity of literature related to LBP education was identified from healthcare professions other than medicine, physiotherapy, chiropractic, and osteopathy; potentially highlighting gaps in other professions in identifying educational requirements related to LBP. The second and third iterations of the LBP-CCS were informed by review from the working group and all members of the working group approved the final iteration.

The LBP-CCS provides LBP educational content that should be feasible to incorporate into entry-level clinical training. Achieving a balance between providing constructive guidelines without dictating how the content should be taught is challenging. Highly prescriptive content recommendations (e.g., do not prescribe opioids in the management of LBP) might hold benefits of greater consistency of content across clinical training programs without individual interpretation. However, the exact recommendations to be included would be difficult to agree upon, would likely be nuanced depending on healthcare profession or geographic region (as seen in clinical guidelines from different regions [5]), and would need to be more frequently updated as specific knowledge evolves. The working group agreed that the content included in the LBP-CCS be less prescriptive to maintain flexibility of use. However, the content of the LBP-CCS is, therefore, more open to individual interpretation. Strategies were included to minimise negative effects of individual interpretation, including: (i) explanation of the need to reflect on current evidence; (ii) the use of explanatory statements to provide context to each topic; and (iii) the provision of high-quality suggested resources to inform use of the LBP-CCS.

### Implementation of the low back pain curriculum content standards

The LBP-CCS have been designed to be used in entry-level clinical training programs for future healthcare clinicians involved in the assessment or management of patients presenting with LBP. The LBP-CCS can be used to guide the development of content in new programs or revise/benchmark content in existing programs. It must be noted that the time available to teach content related to LBP in different clinical training programs may differ considerably, which will impact the degree of detail to which the LBP-CCS can be implemented. For example, in an Australian study the number of hours related to the teaching of spinal assessment and management ranged from 2 h in pharmacy training to 310 h in chiropractic training [12]. In addition, the level of detail required for each item within the LBP-CCS may differ between clinical training programs and healthcare professions. For example, pharmacy programs would need to teach more detail related to the use of pharmaceutical management options for LBP, whereas physiotherapy programs would need to teach more detail on exercise and manual therapy options. Therefore, the LBP-CCS has been designed to provide high-level guidance regarding the content that should be covered, while acknowledging that the implementation of the LBP-CCS within individual academic programs may vary depending upon numerous factors. Moving forward, the development of profession-specific versions of the LBP-CCS, informed by professional representatives, could be considered to identify the content of most importance for each profession, while recognising time restraints within training programs.

Wide-spread dissemination of the LBP-CCS is essential to facilitate global uptake and produce change in LBP education standards. The LBP-CCS and associated resources are freely available online [34] and these will be disseminated to entry-level clinical training programs worldwide. Dissemination will occur through endorsing organisations, including professional organisations with global reach, working group members, and directly to entry-level clinical training programs.

## Conclusion

We have developed the LBP-CCS in consultation with an interdisciplinary, international working group. These standards can be used to develop or benchmark the content of curriculum related to LBP in new or existing entry-level clinical training programs. Use of the LBP-CCS will help to increase the consistency and quality of LBP education.

### Electronic supplementary material

Below is the link to the electronic supplementary material.

Supplementary Material 1

## Data Availability

The datasets used and/or analysed during the current study are available from the corresponding author on reasonable request.

## Abbreviations

LBP: Low back pain
LBP-CCS: Low Back Pain Curriculum Content Standards
ISSLS: International Society for the Study of the Lumbar Spine
IFOMPT: International Federation of Orthopaedic Manipulative Physical Therapists
WP: World Physiotherapy
WFC: World Federation of Chiropractic
EFIC: European Pain Federation
MACP: Musculoskeletal Association of Chartered Physiotherapists
CPDANZ: Council of Physiotherapy Deans Australia and New Zealand
IASP: International Association for the Study of Pain
