# Supplementary material for: Development of low back pain curriculum content standards for entry-level clinical training
Source: BMC Med Educ. 2024 Feb 12;24:136. doi: 10.1186/s12909-024-05086-x (PMC10863179; doi:10.1186/s12909-024-05086-x)
Supplement: Supplementary file 1 — Supplementary Material 1 [file 12909_2024_5086_MOESM1_ESM.docx]

**Additional file 1: Search strategies**

Search strategy – Medline and Embase

1. low back pain.mp. or Back Pain/ or Low Back Pain/

2. Education, Medical/ or Competency-Based Education/ or education.mp. or Education, Medical, Undergraduate/ or Health Education/ or Education/

3. Knowledge/ or Health Knowledge, Attitudes, Practice/ or knowledge.mp.

4. competency.mp.

5. confidence.mp.

6. students.mp. or Students/ or Students, Medical/ or Students, Health Occupations/

7. clinician.mp.

8. General Practitioners/ or practitioner.mp.

9. Allied Health Personnel/ or Allied Health Occupations/ or allied health.mp.

10. physiotherapist.mp. or Physical Therapists/

11. Chiropractic/ or chiropract*.mp.

12. medical.mp.

13. 6 or 7 or 8 or 9 or 10 or 11 or 12

14. learning/ or learning outcomes.mp.

15. curriculum development/ or curriculum/ or curriculum.mp.

16. practice ready.mp. or clinical competence/

17. 2 or 3 or 14 or 15

18. 4 or 5 or 16

19. 1 and 13 and 17 and 18

**Search strategy – PsychInfo**

1 low back pain.mp. or Back Pain/ or Low Back Pain/ 5475

2 Education, Medical/ or Competency-Based Education/ or education.mp. or Education, Medical, Undergraduate/ or Health Education/ or Education/ 536789

3 Knowledge/ or Health Knowledge, Attitudes, Practice/ or knowledge.mp. 357197

4 competency.mp.

5 confidence.mp. 88568

6 students.mp. or Students/ or Students, Medical/ or Students, Health Occupations/ 643296

7 clinician.mp. 32988

8 General Practitioners/ or practitioner.mp. 27615

9 Allied Health Personnel/ or Allied Health Occupations/ or allied health.mp. 4550

10 physiotherapist.mp. or Physical Therapists/ 1084

11 Chiropractic/ or chiropract*.mp. 485

12 medical.mp. 265737

13 6 or 7 or 8 or 9 or 10 or 11 or 12 928266

14 learning/ or learning outcomes.mp. 78108

15 curriculum development/ or curriculum/ or curriculum.mp. 72880

16 practice ready.mp. or clinical competence/ 27

17 2 or 3 or 4 or 5 or 14 or 15 or 16 954255

18 1 and 13 and 20 252

**Search – Cinahl**

S5 S1 AND S2 AND S3 AND S4

S4 "student* OR clinician* OR practitioner OR health OR medical OR chiropract* OR physiotherap* OR "physical therapist"" OR (MH "Schools, Health Occupations+") OR (MH "Students, Allied Health+") OR (MH "Physical Therapists") OR (MH "Students, Chiropractic") OR (MH "Students, Medical") OR (MH "Students, Physical Therapy")

S3 TI, AB competency OR confidence OR "clinical competence" OR "practice ready"

S2 ( TI,AB (education OR knowledge OR learning OR "learning outcome*" OR curriculum OR "curriculum development" OR undergraduate OR postgraduate ) OR MW ( education OR learning OR curriculum OR knowledge )

S1 TI,AB "low back pain" OR MH low back pain

**Additional file 2: Literature used to inform the development of the Low Back Pain Curriculum Content Standards**

**Database search**

1. Allen SS, Bland CJ, Harris IB, Anderson D, Poland G, Satran L, et al. Structured clinical teaching strategy. Medical teacher. 1991;13(2):177-84.

2. Blumenthal D, Gokhale M, Campbell EG, Weissman JS. Preparedness for Clinical Practice. JAMA. 2001;286(9):1027.

3. Briggs AM, Slater H, Smith AJ, Parkin-Smith GF, Watkins K, Chua J. Low back pain-related beliefs and likely practice behaviours among final-year cross-discipline health students. European Journal of Pain. Vol.17(5), 2013, pp. 766-775.; 2013.

4. Brunner E, Dankaerts W, O'Sullivan K, Meichtry A, Bauer C, Probst M. Associations between alliance, physiotherapists' confidence in managing the patient and patient-reported distress in chronic low back pain practice. European Journal of Physiotherapy. 2021;23(3):196-200.

5. Cayea D, Perera S, Weiner DK. Chronic low back pain in older adults: What physicians know, what they think they know, and what they should be taught. Journal of the American Geriatrics Society. 2006;54(11):1772-7.

6. Clawson K, Jackson D, Ostergaard D. It's past time to reform the musculoskeletal curriculum. Academic Medicine. 2001;76(7):709-10.

7. Domenech J, Sanchez-Zuriaga D, Segura-Orti E, Espejo-Tort B, Lison JF. Impact of biomedical and biopsychosocial training sessions on the attitudes, beliefs, and recommendations of health care providers about low back pain: A randomised clinical trial. Pain. Vol.152(11), 2011, pp. 2557-2563.; 2011.

8. Dwyer CP, MacNeela P, Durand H, O'Connor LL, Main CJ, McKenna-Plumley PE, et al. Effects of biopsychosocial education on the clinical judgments of medical students and GP trainees regarding future risk of disability in chronic lower back pain: A randomized control trial. [References]: Pain Medicine. Vol.21(5), 2020, pp. 939-950.; 2020.

9. Elvén M, Dean E. Factors influencing physical therapists’ clinical reasoning: qualitative systematic review and meta-synthesis. Physical Therapy Reviews. 2017;22(1-2):60-75.

10. Fields HL. Core curriculum for professional education in pain., Rev. 1st ed: (1991). Core curriculum for professional education in pain. v, 97 pp. Seattle, WA, US: IASP Press; US.; 1991.

11. French SD, Nielsen M, Hall L, Nicolson PJ, Bennell K, Hinman R, et al. Essential key messages about diagnosis, imaging, and self-care for people with low back pain: a modified Delphi study of consumer and expert opinions. Pain. 2019.

12. Fullen BM, Baxter GD, O'Donovan BGG, Doody C, Daly LE, Hurley DA. Factors impacting on doctors' management of acute low back pain: A systematic review. European Journal of Pain. Vol.13(9), 2009, pp. 908-914.; 2009.

13. Haq I, Fuller J, Dacre J. The use of patient partners with back pain to teach undergraduate medical students. Rheumatology. 2006;45(4):430-4.

14. Hoeger Bement MK, Sluka KA. The Current State of Physical Therapy Pain Curricula in the United States: A Faculty Survey. The Journal of Pain. 2015;16(2):144-52.

15. Innes SI, Leboeuf-Yde C, Walker BF. Chiropractic student choices in relation to indications, non-indications and contra-indications of continued care. Chiropractic & manual therapies. 2018;26:3.

16. Latimer J, Maher C, Refshauge K. The Attitudes and Beliefs of Physiotherapy Students to Chronic Back Pain. The Clinical Journal of Pain. Vol.20(1), 2004, pp. 45-50.; 2004.

17. Learman KE, Ellis AR, Goode AP, Showalter C, Cook CE. Physical therapists' clinical knowledge of multidisciplinary low back pain treatment guidelines. Physical therapy. 2014;94(7):934-46.

18. Licciardone JC. A National Study of Primary Care Provided by Osteopathic Physicians. The Journal of the American Osteopathic Association. 2015;115(12):704-13.

19. Mezei L, Murinson BB. Pain Education in North American Medical Schools. The Journal of Pain. 2011;12(12):1199-208.

20. Murinson B, Rosenberg J. The new pain curriculum: A guide for neurologists who teach. Nature Clinical Practice Neurology. 2008;4(11):634-5.

21. Murray TJ. Concepts in undergraduate neurological teaching. Clinical Neurology and Neurosurgery. 1977;79(4):273-84.

22. Nyiendo JA, Haldeman S. A critical study of the student interns' practice activities in a chiropractic college teaching clinic. Journal of manipulative and physiological therapeutics. 1986;9(3):197-207.

23. Oza SK, Joo P, Grochowalski JH, Rougas S, George P, Milan F. Novel use of an osce to assess medical students' responses to a request for a low value diagnostic imaging test: A mixed methods analysis: Patient Education and Counseling. 2021, pp. No Pagination Specified.; 2021.

24. Ryan C, Murphy D, Clark M, Lee A. The effect of a physiotherapy education compared with a non-healthcare education on the attitudes and beliefs of students towards functioning in individuals with back pain: an observational, cross-sectional study. Physiotherapy. 2010;96(2):144-50.

25. Scudds RJ, Scudds RA, Simmonds MJ. Pain in the physical therapy (pt) curriculum: a faculty survey. Physiotherapy Theory and Practice. 2001;17(4):239-56.

26. Shipton EE, Bate F, Garrick R, Steketee C, Shipton EA, Visser EJ. Systematic Review of Pain Medicine Content, Teaching, and Assessment in Medical School Curricula Internationally. Pain and Therapy. 2018;7(2):139-61.

27. Shipton EE, Steketee C, Bate F, Visser EJ. Exploring assessment of medical students' competencies in pain medicine-A review. The Pain Report. 2019;4(1):e704.

28. Simmonds MJ, Derghazarian T, Vlaeyen JWS. Physiotherapists' knowledge, attitudes, and intolerance of uncertainty influence decision making in low back pain. The Clinical Journal of Pain. Vol.28(6), 2012, pp. 467-474.; 2012.

29. Slater H, Jordan JE, O'Sullivan PB, Schütze R, Goucke R, Chua J, et al. “Listen to me, learn from me”: a priority setting partnership for shaping interdisciplinary pain training to strengthen chronic pain care. Pain. 2022:10.1097.

30. St-Onge C, Martineau B, Harvey A, Bergeron L, Mamede S, Rikers R. From See One Do One, to See a Good One Do a Better One: Learning Physical Examination Skills Through Peer Observation. Teaching and Learning in Medicine. 2013;25(3):195-200.

31. Steiner BD, Cook RL, Smith AC, Curtis P. Does training location influence the clinical skills of medical students? Academic Medicine. 1998;73(4):423-6.

32. Weiner DK, Morone NE, Spallek H, Karp JF, Schneider M, Washburn C, et al. E-Learning Module on Chronic Low Back Pain in Older Adults: Evidence of Effect on Medical Student Objective Structured Clinical Examination Performance. Journal of the American Geriatrics Society. 2014;62(6):1161-7.

33. Widerstrom B, Rasmussen-Barr E, Bostrom C. Aspects influencing clinical reasoning and decision-making when matching treatment to patients with low back pain in primary healthcare. Musculoskeletal Science & Practice. 2019;41:6-14.

34. Wiest FC, Ferris TG, Gokhale M, Campbell EG, Weissman JS, Blumenthal D. Preparedness of internal medicine and family practice residents for treating common conditions. JAMA. 2002;288(20):2609-14.

**Guideline documents**

1. Oliveira CB, Maher CG, Pinto RZ, Traeger AC, Lin C-WC, Chenot J-F, et al. Clinical practice guidelines for the management of non-specific low back pain in primary care: an updated overview. European Spine Journal. 2018:1-13.

2. Philippine Academy of Rehabilitation Medicine. Clinical Practice Guidelines on the Diagnosis and Management of Low Back Pain Updated 2017. Accessed March, 2022. Available from: https://parm.org.ph/pdf/lbp.pdf.

3. Toward Optimized Practice. Evidence-informed primary care management of low back pain. Clinical practice guideline 3rd Ed. 2017 Accessed March, 2022. Available from: https://actt.albertadoctors.org/media/zpgdhot5/lbp-guideline.pdf?_20180625085852

**Accreditation documents**

1. Standards for assessment and accreditation of primary medical programs by the Australian Medical Council 2012. Australian Medical Council Limited; 2012.

2. Physiotherapy practice thresholds in Australia and Aotearoa New Zealand. Physiotherapy Board of Australia and Physiotherapy Board of New Zealand; 2015.

3. Accreditation standards for chiropractic programs Competency standards for graduating chiropractors. Council on Chiropractic Education Australasia; 2017.

4. Entry-to-practice competency profile for chiropractors in Canada. Council on Chiropractic Education Canada, Federation of Canadian Chiropractic; 2018.

5. Accreditation procedures and standards in first qualification chiropractic education and training. European Council on Chiropractic Education; 2019.

6. National physiotherapy entry-to-practice curriculum guidelines. Canadian Council of Physiotherapy University Programs; 2019.

7. Standards and required elements for accreditation of physical therapist education programs. Commission on Accreditation in Physical Therapy Education; 2020.

8. Basic medical education WFME global standards for quality improvement. World Federation for Medical Education; 2020.

9. CCE accreditation standards. Council on Chiropractic Education; 2021.

10. CACMS standards and elements: standards for accreditation of medical education programs leading to the MD degree. Committee on Accreditation of Canadian Medical Schools; 2021.

**Working group recommendations**

1. Global competency and outcomes framework for universal health coverage. Geneva: World Health Organization 2022.

2. The pain management core curriculum for European medical schools. Committee on Education for the European Federation of IASP Chapters; 2013.

3. Core curriculum for the European diploma in pain medicine. European Pain Federation; 2016.

4. Core curriculum for the European diploma in pain physiotherapy. European Pain Federation; 2017.

5. Core curriculum for the European diploma pain psychology. European Pain Federation; 2019.

6. Core curriculum for the European diploma in pain nursing. European Pain Federation; 2019.

7. Education policy statement. World Physiotherapy; 2019.

8. Australian Commission on Safety and Quality in Health Care. Low back pain clinical care standard. Sydney: ACSQHC; 2022.

9. Buchbinder R, van Tulder M, Öberg B, Costa LM, Woolf A, Schoene M, et al. Low back pain: a call for action. The Lancet. 2018;391(10137):2384-8.

10. Chehade MJ, Burgess TA, Bentley DJ. Ensuring quality of care through implementation of a competency‐based musculoskeletal education framework. Arthritis Care & Research. 2011;63(1):58-64.

11. Foster NE, Anema JR, Cherkin D, Chou R, Cohen SP, Gross DP, et al. Prevention and treatment of low back pain: evidence, challenges, and promising directions. The Lancet. 2018.

12. Hartvigsen J, Hancock MJ, Kongsted A, Louw Q, Ferreira ML, Genevay S, et al. What low back pain is and why we need to pay attention. The Lancet. 2018.

13. Health Education England and NHS England. Musculoskeletal core capabilities framework for first point of contact practitioners. England; 2018.

14. Maher C, Underwood M, Buchbinder R. Non-specific low back pain. The Lancet. 2017;389(10070):736-47.

15. Royal College of General Practitioners. Clinical topic guides: Musculoskeletal health 2022 [Available from: https://www.rcgp.org.uk/training-exams/training/gp-curriculum-overview/clinical-topic-guides#musculoskeletal.

16. Task Force on Professional Education. Core curriculum for professional education in pain. International Association for the Study of Pain, editor. Seattle: IASP Publications; 1991.

17. IASP interprofessional psin curriculum outline taskforce. IASP Interprofessional pain curriculum outline: International Association for the Study of Pain; 2012 [Available from: https://www.iasp-pain.org/education/curricula/iasp-interprofessional-pain-curriculum-outline/.

**Additional file 3: Low Back Pain Curriculum Content Standards**

**PREAMBLE**

Low back pain is one of the leading causes of disability world-wide and has significant societal impact due to the high prevalence and associated lost productivity and healthcare costs. The management of low back pain is highly variable. Many patients do not receive recommended care and/or they receive care that is not recommended by clinical practice guidelines. Graduating healthcare professionals who care for people with low back pain require knowledge of current best-evidence management of low back pain. However, currently there are no clear educational standards for low back pain across healthcare training programs. The Low Back Pain Curriculum Content Standards (LBP-CCS) reported in this document have been designed to increase the consistency and quality of low back pain education.

The LBP-CCS have been developed through a consensus process by an international, interdisciplinary working group of researchers, academics, clinicians (including representatives from physiotherapy, chiropractic, medicine, psychology, pharmacy, and osteopathy), and healthcare consumers. The LBP-CCS are intended to be used to guide curriculum content in academic programs providing entry-level education for healthcare professionals who will be involved in the assessment and/or management of people with low back pain in primary care. This may include undergraduate or postgraduate programs which enable the graduating student to be registered as a healthcare professional. Other groups, such as accreditation bodies, policy-makers, and students may also use this document to inform them of current low back pain educational standards.

The LBP-CCS can be used to help develop new programs or as a benchmark to map against content in existing programs. Current evidence is continually evolving, clinical practice may differ due to local context and cultural considerations, and the level/detail of required knowledge may be different for different academic programs. Therefore, the LBP-CCS have been designed to provide high-level guidance to the content that should be covered in an academic program. It is expected that individual programs incorporate the content into the curriculum, after considering: (i) the current evidence-base; (ii) the level of detail required for the program; and (iii) the local context.

The LBP-CCS, while focused on low back pain, are intended to be integrated into an academic program in conjunction with generic competencies required for patient assessment and management, such as: person-centred care, shared decision making, effective communication skills; clinical reasoning; knowledge of local culture, healthcare systems, and policy; and principles of evidence-based practice. The LBP-CCS will undergo periodic review to accommodate developments in the field and feedback from users.

The LBP-CCS is divided into four sections:

1. Objectives: Overarching objectives to be achieved by using the LBP-CCS. It is not intended that these replace existing learning objectives of individual academic programs.
2. Content outline: Four broad content topics, each with a list of knowledge items and competencies, that should be addressed in a comprehensive curriculum for future health care professionals who will manage patients with low back pain in primary care.
3. Glossary: Definitions for the terminology used within this document
4. Suggested resources: List of suggested resources reflecting current best evidence for low back pain epidemiology, assessment, and management.

**OBJECTIVES**
Using the LBP-CCS will help ensure that students graduating from entry-level education for healthcare professionals can:

1: Describe the epidemiology and public health impact of low back pain based on the current evidence. Explain the burden associated with low back pain in a local and global context, and with consideration of different populations.

2: Apply the biopsychosocial model to assess individuals with low back pain to: (i) classify individual low back pain presentations and exclude serious underlying pathology; and (ii) identify relevant contributors to acute, sub-acute, persistent or recurrent pain and disability.

3: Communicate effectively with the patient and other support figures (e.g., family, workplace, health care providers, insurers) in a dialogue about the low back pain presentation (including patient beliefs, goals, and concerns), prognosis, and the proposed management plan.

4: Develop and implement a safe and effective person-centred management plan that addresses the individual’s goals and preferences, is based on the patient’s individual assessment findings and the best available evidence, considers cultural and biopsychosocial factors, and includes the development of effective self-management strategies.

5: Evaluate the patient’s progress using appropriate outcome measures to assess individual patient goals and adapt the management plan accordingly, including providing referral to other healthcare practitioners as appropriate.

6. Describe the evidence behind primary, secondary, and tertiary prevention strategies and how to support low back pain prevention in healthcare settings and the community, including self-management strategies.

**CONTENT OUTLINE**Below is the content that students should learn in a comprehensive curriculum for future healthcare practitioners who will manage people with low back pain. The content is divided into knowledge and competencies under four topics. Under each topic is an explanatory statement describing the importance and relevance of the topic. Knowledge provides an outline of the key information students need to learn throughout the academic program. Competencies outline the key capabilities graduating health care professionals need to demonstrate by the end of their academic training program.

**Topic 1. Epidemiology of low back pain and the public health impact**Understanding the epidemiology of low back pain and the public health impact, including etiology, prevalence, prognosis, and burden, informs the provision of high-quality care for individuals with low back pain and low back pain prevention strategies.

1.1 Knowledge

- Challenges in determining a specific source of pain for the majority of low back pain presentations
- Classification of low back pain and how classification informs appropriate management, including:
  - Serious spinal pathology (e.g., cancer, infection, cauda equina syndrome, fracture)
  - Specific spinal pathology (e.g., nerve root involvement (radicular pain and radiculopathy), spinal stenosis, inflammatory arthritis, osteoporosis)
  - Referred pain from non-spinal pathology (e.g., abdominal aortic aneurysm, kidney pathology)
  - Non-specific low back pain
- Etiology and pathophysiology of low back pain across the lifespan and within different population groups (e.g., children, older adults, pregnancy-related back pain)
- Clinical course and trajectories of low back pain across the lifespan and within different population groups, including:
  - Prevalence and incidence of new episodes
  - Recovery from an episode
  - Recurrent episodes and/or flare-ups
  - Development of persistent pain
- The burden and public health impact of low back pain from a local and global perspective, including:
  - Quality of life, ability to work, social function, psychological impact
  - Disability and sickness benefits, lost productivity
  - Costs to the healthcare system
  - Health and social inequities
  - Increased burden and costs from low value low back pain management
- Historical perspectives related to the management of low back pain and the relationship with current low back pain burden to individuals and healthcare systems
- Public health and community strategies to decrease the incidence and pain-related disability of low back pain (primary prevention) and promote self-management (secondary and tertiary prevention)

1.2 Competencies

- Discuss the challenges of determining a specific source of pain for low back pain presentations
- Explain the role of using a classification system to inform the appropriate management of low back pain
- Describe serious and specific pathologies that may present as low back pain across the lifespan
- Incorporate knowledge of low back pain etiology, prevalence, and prognosis into the clinical reasoning process for an individual patient
- Explain the burden of low back pain and the importance of optimal strategies to prevent and manage low back pain in the community

**Topic 2. Biopsychosocial contributors to the development and course of low back pain**Current evidence demonstrates the importance of evaluating and managing low back pain using a biopsychosocial model to explore various biological, psychological, and social contributors to the development and course of low back pain

2.1 Knowledge

- Pain science related to the multidimensional nature of pain, including:
  - Pain mechanisms and manifestations (e.g., nociceptive, nociplastic, peripheral and central neuropathic, peripheral and central sensitisation, allodynia, hyperalgesia)
  - Dynamic interplay and impact of biological, psychological, and social contributors to the development, experience, and course of pain and related disability
- Immunological, endocrine, and neurological responses to intrinsic (e.g., ageing) and extrinsic (e.g., tissue injury, stress) stimuli, including the stages of tissue healing/repair and degeneration
- Musculoskeletal and neurological anatomy, function, and biomechanics of the lumbar spine and pelvis
- Validity and clinical relevance of potential biological contributors to the development and course of low back pain and related disability across the lifespan, including:
  - Potential nociceptive sources: muscles; bones; joints; ligaments; discs; nerves; spinal cord and dura
  - Body structure and function: postural change/spinal deformity (e.g., scoliosis, spondylolisthesis, vertebral compression, pregnancy); strength, flexibility, and coordination; mechanical or repetitive loads
  - Inflammatory changes and tissue healing/repair
  - Degenerative changes
  - Genetics
  - Lifestyle factors: physical activity levels; physical stress; sleep quality/disturbance; obesity; smoking; medication
  - General health: other pains/multi-site pain; other health conditions/co-morbidities; pro-nociceptive/pro-inflammatory states
- Validity and clinical relevance of potential psychological contributors to the development, experience, and course of low back pain and related disability across the lifespan, including:
  - Beliefs about the underlying nature of pain and activity
  - Expectations about pain, recovery, and treatment options
  - Worry and pain catastrophising
  - Pain self-efficacy
  - Pain coping
  - Depression and low mood
  - Psychological stress, anxiety, and pain-related fear
- Validity and clinical relevance of potential social contributors to the development, experience, and course of low back pain and related disability across the lifespan, including:
  - Beliefs and attitudes of relevant others (e.g., friends, family, work colleagues)
  - Background, culture, and values
  - Perceived social support and isolation
  - Employment and work environment including job satisfaction
  - Socioeconomic factors: income/wealth/poverty; healthcare access; level of education; health literacy, neighbourhood and built environment

2.2 Competencies

- Describe the multifactorial nature of the development, experience, and course of low back pain and related disability, including biological, psychological, and social contributors
- Evaluate current evidence for the validity and clinical relevance of a wide range of potential biological, psychological, and social contributors to the development and course of low back pain, including the transition from acute to persistent pain
- Identify features of different pain mechanisms and manifestations
- Incorporate knowledge of biological, psychological, and social contributors into the clinical reasoning process for an individual patient

**Topic 3. Clinical assessment and investigations**An individualised, person-centred approach to low back pain clinical assessment, based on the best available evidence and incorporating effective communication skills, is recommended to: (i) determine patient beliefs, concerns, and goals; (ii) identify important contributors to low back pain; (iii) guide low back pain classification/diagnosis; and (iv) develop an appropriate individualised management plan

3.1 Knowledge

- Key information that should be collected during the patient history, including:
  - Current presentation, including symptoms and impairments
  - How pain impacts the patient’s life
  - Previous medical history, including relationship with pain
  - Patient beliefs, concerns, health confidence, and expectations/goals of care
  - Relevant social and family history
- Validity, reliability, interpretation, and appropriate use of patient reported outcome measures (e.g., Numerical Rating Scale, Oswestry Disability Index)
- Validity, reliability, interpretation, and appropriate use of risk assessment and risk stratification tools (e.g., STarT Back tool, Orebro musculoskeletal pain screening questionnaire)
- Validity, reliability, interpretation, and appropriate use of elements of the clinical assessment to classify/diagnose the low back pain presentation, including:
  - Patient history information
  - Red flags to identify potential serious pathology and when to escalate them
  - Physical examination tests
- Validity, reliability, interpretation, appropriate use, and risks and benefits of performing diagnostic investigations (e.g., medical imaging, blood tests)
- Impact of cultural, societal, and economic influences on the optimal assessment of low back pain

3.2 Competencies

- Perform a person-centred patient history and physical examination guided by effective clinical reasoning and best-evidence
- Identify the patient’s primary concerns and underlying beliefs about pain, pain management, and expectations of recovery
- Identify the patient’s goals of care
- Identify patients at high risk of recurrent or persistent low back pain
- Consider the role of presenting symptoms, impairments, and psychological influences on functional limitations and participation restrictions
- Discuss with the patient whether referral for investigations is needed to direct appropriate management, including discussion of the related risks and benefits
- Interpret and communicate investigation findings to the patient in the context of their individual clinical presentation, and in a manner that minimises potential risks (e.g., worry/concern from incidental findings)
- Synthesise clinical assessment and, where relevant, investigation findings and discuss these with the patient in a manner that provides a meaningful explanation of their low back pain and addresses their beliefs, expectations, and concerns
- Identify patients with potential serious pathology (e.g., cancer, cauda equina syndrome, infection, fracture)

**Topic 4. Developing a clinical management plan for low back pain**
A person-centred approach, utilising current best-evidence and shared-decision making, is recommended to develop an individualised management plan to address the goals and priorities of the patient and support the patient in developing self-management skills

4.1 Knowledge

- Current best evidence of the effectiveness, cost-effectiveness, contraindications/indications/non-indications, and benefits/risks of potential interventions for the management of low back pain including:
  - Education/advice: personalised reassurance to address individual concerns; pain education to address unhelpful beliefs; return to work/normal daily activities; increasing general physical activity/reducing sedentary behaviour; self-management advice
  - Exercise approaches: cardiovascular, strength; flexibility; motor control; graded activity
  - Manual therapy: joint manipulation/mobilisation; massage
  - Pharmacological management: NSAIDs; paracetamol; muscle relaxants; antidepressants; weak and strong opioids
  - Psychological therapy (e.g., cognitive behavioural therapy)
  - Adjunct modalities (e.g., heat/ice, electrophysical therapy, ergonomic devices, emerging technology, clinical decision support tools)
  - Complementary therapies (e.g., acupuncture, herbal medicine)
  - Injections: pharmacological (e.g., steroids); non-pharmacological (e.g., stem cells)
  - Interventional procedures (e.g., radiofrequency denervation)
  - Surgery (e.g., decompression, fusion) and pre- and post-surgical rehabilitation
- Appropriate progression of interventions, based on careful reassessment and considering the individual patient goals
- The healthcare professionals who are qualified to perform different interventions to manage low back pain, and appropriate referral pathways in the local healthcare system
- How to perform specific interventions within scope of care
- Current clinical practice guideline recommendations for the management of low back pain (acute/recurrent/persistent)
- Strategies to promote self-management including: advice/education/reassurance; behavioural change techniques; health coaching designed to increase uptake of active behaviours; and use of eHealth innovations to support this
- Occupational health considerations, including: return to work support; modified activities; local policies and procedures
- Influence of natural history and contextual factors (e.g., features of the patient-therapist relationship) on patient recovery

4.2 Competencies

- Use shared decision making and a person-centred approach to develop an individualised evidence-based management program, including consideration of:
  - Likely natural course of the low back pain presentation and related disability
  - Classification of the presentation, including consideration of pain duration (acute/recurrent/persistent)
  - Identified biopsychosocial contributors
  - Intervention options for the low back pain presentation, including discussion of related risks and benefits
  - Appropriate progression of interventions
  - Patient preferences and goals of care
- Support the patient to reach an informed decision about their management program
- Provide individualised advice/education/reassurance/health coaching to improve patient understanding about their low back pain presentation, reduce patient concerns, and encourage self-management and active behaviours
- Provide high quality delivery of selected interventions within scope of care
- Identify and provide timely referral when patients require further assessment or treatment from other healthcare professionals
- Collaborate effectively as part of an interdisciplinary team
- Communicate and collaborate with occupational health providers (including insurance agencies) and the workplace to facilitate return to work and appropriate work activities
- Discuss the importance of self-management and provide self-management strategies/resources to help prevent or manage recurrent or persistent low back pain episodes or flare-ups
- Regularly assess patient progress towards individual goals of management using appropriate outcome measures and discuss potential changes to the management plan with the patient

**GLOSSARY**

- **Biopsychosocial contributors:** The biological/physical, psychological, and social factors that contribute to the onset and/or course of pain and disability experienced by people with low back pan
- **Classification:** Classification of the clinical presentation into meaningful categories based on a person’s signs and symptoms that may help to explain the nature of the presentation and/or direct appropriate management
- **Clinical assessment:** Assessment of the patient, including patient history and physical examination, to determine a provisional classification/diagnosis
- **Clinical reasoning:**
  (1) “The thinking or reasoning that a health practitioner engages in to solve and manage a clinical problem.”

Mapping clinical reasoning literature across the health professions: a scoping review

*https://bmcmededuc.biomedcentral.com/articles/10.1186/s12909-020-02012-9*

(2) “Gathering and synthesizing information; generating hypotheses; and formulating a clinical impression, prognosis, diagnosis, treatment, care, and/or management plan”

Drawing Boundaries: The Difficulty in Defining Clinical Reasoning

*https://journals.lww.com/academicmedicine/Fulltext/2018/07000/Drawing_Boundaries__The_Difficulty_in_Defining.17.aspx*

- **Diagnosis:** The process of determining which disease, condition, or injury explains a person’s signs and symptoms. Determining a diagnosis may help direct appropriate management for the patient.
- **Evidence-based practice:** “The integration of the best available research evidence with clinical expertise and the patient’s unique values and circumstances”

Straus, S. E., Glasziou, P., Richardson, W. S., & Haynes, R. B. (2011). Evidence-based medicine: How to practice and teach it (4th ed.). Churchill Livingstone Elsevier.

- **Interventions:** A treatment, procedure, or other action taken to prevent or treat disease, or improve health.
- **Investigations:** A diagnostic test used to help confirm/refute a serious/specific diagnosis (e.g., diagnostic imaging, laboratory tests). Investigations may be indicated after a thorough clinical assessment when serious or specific pathology is suspected.
- **Management plan:** The individualised plan, discussed and agreed upon with the patient, to manage the patient’s low back pain presentation and address their goals of care. Includes information about prognosis, selected interventions with appropriate progressions, referral/co-management as required, and reassessment.
- **Non-specific low back pain:** Low back pain presentations where it is not possible to identify a specific or serious pathology as the cause of the pain.
- **Occupational health:** Providing health care to workers with work-related injuries or illnesses and working with insurers, businesses, and regulators to facilitate return to work and create safer workplaces
- **Patient history:** The patient’s subjective report of their presenting condition and relevant biopsychosocial contributors and previous health history
- **Patient reported outcome measures (PROMs):** “Questionnaires which capture a person’s perception of their own health. They enable patients to report on their quality of life, daily functioning, symptoms, and other aspects of their health and well-being.”

Australian Commission on Safety and Quality in Healthcare

*https://www.safetyandquality.gov.au/our-work/indicators-measurement-and-reporting/patient-reported-outcome-measures#:~:text=Patient%2Dreported%20outcome%20measures%20(PROMs,their%20health%20and%20well%2Dbeing*.

- **Person-centred care:** “The person-centred approach treats each person respectfully as an individual human being, and not just as a condition to be treated. It involves seeking out and understanding what is important to the patient, their families, carers and support people, fostering trust and establishing mutual respect. It also means working together to share decisions and plan care.”

Australian Commission on Safety and Quality in Healthcare *https://www.safetyandquality.gov.au/our-work/partnering-consumers/person-centred-care*

- **Physical examination:** An evaluation of the body and its function using observation, palpation, orthopaedic, neurological, and functional tests
- **Prevention, primary:** “Primary prevention aims to prevent disease or injury before it ever occurs. This is done by preventing exposures to hazards that cause disease or injury, altering unhealthy or unsafe behaviours that can lead to disease or injury, and increasing resistance to disease or injury should exposure occur.”

Institute for Work & Health
*https://www.iwh.on.ca/what-researchers-mean-by/primary-secondary-and-tertiary-prevention*

- **Prevention, secondary:** “Secondary prevention aims to reduce the impact of a disease or injury that has already occurred. This is done by detecting and treating disease or injury as soon as possible to halt or slow its progress, encouraging personal strategies to prevent reinjury or recurrence, and implementing programs to return people to their original health and function to prevent long-term problems.”

Institute for Work & Health
*https://www.iwh.on.ca/what-researchers-mean-by/primary-secondary-and-tertiary-prevention*

- **Prevention, tertiary:** “Tertiary prevention aims to soften the impact of an ongoing illness or injury that has lasting effects. This is done by helping people manage long-term, often-complex health problems and injuries (e.g., chronic diseases, permanent impairments) in order to improve as much as possible their ability to function, their quality of life and their life expectancy.”

Institute for Work & Health
*https://www.iwh.on.ca/what-researchers-mean-by/primary-secondary-and-tertiary-prevention*

- **Red flags:** Signs and symptoms which indicate the possibility of an underlying serious pathology (e.g., fracture, cancer, infection, cauda equina syndrome)
- **Risk assessment/stratification tools:** “Questionnaires which assess the risk of a patient for developing persistent pain (e.g., Orebro questionnaire) or use risk factors to stratify a patient into appropriate management (e.g., STarT Back tool)
- **Self-management:** “An individual's ability to manage the symptoms, treatment, physical and psychological consequences, and lifestyle changes inherent in living with a condition.”

Self-management at the core of back pain care: 10 key points for clinicians

*https://www.ncbi.nlm.nih.gov/pmc/articles/PMC8353288/*

- **Serious pathology:** Underlying pathology related to the low back pain presentation that needs rapid diagnosis and management to decrease morbidity/mortality (e.g., cancer, infection, cauda equina syndrome, fracture)
- **Shared decision making:** “Shared decision making involves discussion and collaboration between a consumer and their healthcare provider. It is about bringing together the consumer's values, goals and preferences with the best available evidence about benefits, risks and uncertainties of treatment, in order to reach the most appropriate healthcare decisions for that person.”

Australian Commission on Safety and Quality in Healthcare *https://www.safetyandquality.gov.au/our-work/partnering-consumers/shared-decision-making*

- **Specific pathology:** Underlying pathology related to the low back pain presentation where diagnosis may lead to a specific change in management (e.g., nerve root involvement (radicular pain and radiculopathy), spinal stenosis, inflammatory arthritis, osteoporosis)

**SUGGESTED RESOURCES**

1. Lancet low back pain series
   - Hartvigsen J, Hancock MJ, Kongsted A, Louw Q, Ferreira ML, Genevay S, et al. What low back pain is and why we need to pay attention. The Lancet. 2018.
     *https://pubmed.ncbi.nlm.nih.gov/29573870/*
   - Foster NE, Anema JR, Cherkin D, Chou R, Cohen SP, Gross DP, et al. Prevention and treatment of low back pain: evidence, challenges, and promising directions. The Lancet. 2018.
     *https://pubmed.ncbi.nlm.nih.gov/29573872/*
   - Buchbinder R, van Tulder M, Öberg B, Costa LM, Woolf A, Schoene M, et al. Low back pain: a call for action. The Lancet. 2018;391(10137):2384-8
     *https://pubmed.ncbi.nlm.nih.gov/29573871/*
2. Diagnosis and management of low back pain: Traeger A, Buchbinder R, Harris I, Maher C. Diagnosis and management of low-back pain in primary care. CMAJ: Canadian Medical Association journal. 2017;189(45):E1386-E95.
   *https://pubmed.ncbi.nlm.nih.gov/29133540/*
3. Clinical practice guidelines
   - Guideline summary paper: Oliveira CB, Maher CG, Pinto RZ, Traeger AC, Lin C-WC, Chenot J-F, et al. Clinical practice guidelines for the management of non-specific low back pain in primary care: an updated overview. European Spine Journal. 2018:1-13.
     *https://pubmed.ncbi.nlm.nih.gov/29971708/*
   - American College of Physicians low back pain clinical practice guidelines:
     Qaseem A, Wilt TJ, McLean RM, Forciea MA. Noninvasive treatments for acute, subacute, and chronic low back pain: a clinical practice guideline from the American College of Physicians. Annals of Internal Medicine. 2017;166(7):514-30.
     *https://pubmed.ncbi.nlm.nih.gov/28192789/*
   - NICE guidelines: National Institute for Health and Care Excellence. Non-specific low back pain and sciatica: management. NICE guideline NG59. 2016.
     *https://www.nice.org.uk/guidance/ng59*
   - NASS guidelines: North American Spine Society. Evidence-Based Clinical Guidelines for Multidisciplinary Spine Care: Diagnosis & Treatment of Low Back Pain. 2020
     *https://www.spine.org/Research-Clinical-Care/Quality-Improvement/Clinical-Guidelines*
   - Academy of Orthopaedic Physical Therapy of the American Physical Therapy Association: Steven Z. George, Julie M. Fritz, Sheri P. Silfies, Michael J. Schneider, Jason M. Beneciuk, Trevor A. Lentz, John R. Gilliam, Stephanie Hendren, and Katherine S. Norman. Interventions for the Management of Acute and Chronic Low Back Pain: Revision 2021. Journal of Orthopaedic & Sports Physical Therapy 2021 51:11, CPG1-CPG60
     *https://pubmed.ncbi.nlm.nih.gov/34719942/*
   - Occupational health guidelines: Luites JWH, Kuijer PPFM, Hulshof CTJ, Kok R, Langendam MW, Oosterhuis T, Anema JR, Lapré-Utama VP, Everaert CPJ, Wind H, Smeets RJEM, van Zaanen Y, Hoebink EA, Voogt L, de Hoop W, Boerman DH, Hoving JL. The Dutch Multidisciplinary Occupational Health Guideline to Enhance Work Participation Among Low Back Pain and Lumbosacral Radicular Syndrome Patients. J Occup Rehabil. 2022 Sep;32(3):337-352
     *https://pubmed.ncbi.nlm.nih.gov/34313903/*
4. Clinical care standards: Australian Commission on Safety and Quality in Health Care. Low back pain clinical care standard. *https://www.safetyandquality.gov.au/standards/clinical-care-standards/low-back-pain-clinical-care-standard.*
5. WHO classifications of function, diagnosis, and healthcare interventions: *https://www.who.int/standards/classifications*
6. Low back pain messages: French SD, Nielsen M, Hall L, Nicolson PJ, Bennell K, Hinman R, et al. Essential key messages about diagnosis, imaging, and self-care for people with low back pain: a modified Delphi study of consumer and expert opinions. Pain. 2019.
   *https://pubmed.ncbi.nlm.nih.gov/31356451/*
7. Person-centred care: Håkansson Eklund J, Holmström IK, Kumlin T, Kaminsky E, Skoglund K, Höglander J, et al. “Same same or different?” A review of reviews of person-centered and patient-centered care. Patient Education and Counseling. 2019;102(1):3-11.
   *https://pubmed.ncbi.nlm.nih.gov/30201221/*
8. Self-management: Kongsted A, Ris I, Kjaer P, Hartvigsen J. Self-management at the core of back pain care: 10 key points for clinicians. Braz J Phys Ther. 2021 Jul-Aug;25(4):396-406.
   *https://pubmed.ncbi.nlm.nih.gov/34116904/*
9. Patient education resources
   - Low back pain management booklet: *https://www.mq.edu.au/research/research-centres-groups-and-facilities/groups/spinal-pain-research-group/low-back-pain-management-booklet*
   - Low back pain information: *https://painhealth.csse.uwa.edu.au/pain-module/low-back-pain/*
   - Chronic pain information: *https://healthtalk.org/chronic-pain/overview*
